# Acknowledgment to Reviewers of *Clocks & Sleep* in 2021

**DOI:** 10.3390/clockssleep4010002

**Published:** 2022-01-27

**Authors:** Clocks & Sleep Editorial Office

**Affiliations:** MDPI AG, St. Alban-Anlage 66, 4052 Basel, Switzerland

Rigorous peer-reviews are the basis of high-quality academic publishing. Thanks to the great efforts of our reviewers, *Clocks & Sleep* was able to maintain its standards for the high quality of its published papers. Thanks to the contribution of our reviewers, in 2021, the median time to first decision was 21 days and the median time to publication was 49 days. The editors would like to extend their gratitude and recognition to the following reviewers for their precious time and dedication, regardless of whether the papers they reviewed were finally published:
Abhishek ChatterjeeIrene Torres-SánchezAdela BadauJames OlceseAdriano BarraJos H.T. RohlingAlvaro Lopez-SamanesJuan Pedro MartínezAndrew N. CooganKai SpiegelhalderAngelina MaricKatarzyna BurekAnjolii DiazKeith PecorAnna BrzeckaKelly BulkeleyAnton KutikhinKonstantinos KompotisArcady PutilovLauren DoumaArlener D. TurnerLorenzo TonettiAtul Kumar PandeyMaría Del Carmen Pérez-FuentesAxel SteigerMaría M. Morales Suárez-VarelaBeata Greb-MarkiewiczMartina PavlíkováBernard PossidenteMasaru TanakaBharath AnanthasubramaniamMassimo BracciChanpreet SinghMichael SchredlChristina SchmidtMikhail F. BorisenkovConcettina FengaMirja TenhunenDaniel ErlacherM.S. ZobaerDhananjay YadavNeeraj SoniDylan SeltermanPatricia L. Lakin-ThomasFabio FabbianPedro Gil-MadronaFrancesca ConteQian XiaoGaetano RiemmaRoberto FrauGina Maria CaetanoRoger HoGiulio BernardiSerge BrandGiuseppe GrossoSoraya L. VallesGiuseppe SantarpinoYuki MatsumotoHalszka OginskaZurab K. SilagadzeHelena Martynowicz


